# An Easy-To-Use Combination Four-Terminal-Pair/Two-Terminal-Pair AC Transformer Bridge

**DOI:** 10.6028/jres.103.010

**Published:** 1998-04-01

**Authors:** A. Jeffery, J. Q. Shields, L. H. Lee

**Affiliations:** National Institute of Standards and Technology, Gaithersburg, MD 20899-0001

**Keywords:** ac bridge, four-terminal-pair bridge, four-terminal-pair measurements, precision impedance measurements, two-terminal-pair bridge

## Abstract

A new four-terminal-pair bridge, capable of achieving a relative standard uncertainty of 1×10^−9^, was constructed at the National Institute of Standards and Technology by converting a two-terminal-pair bridge. The conversion requires only the addition of components which are easily removed if two-terminal-pair measurements are to be made. The design and testing of this bridge is described. The new four-terminal-pair bridge requires fewer auxiliary balances than the present four-terminal-pair bridge employed at NIST, which makes it much easier to use. This new design can be used to compare capacitance, resistance, and inductance standards.

## 1. Introduction

**Fig. 1:**
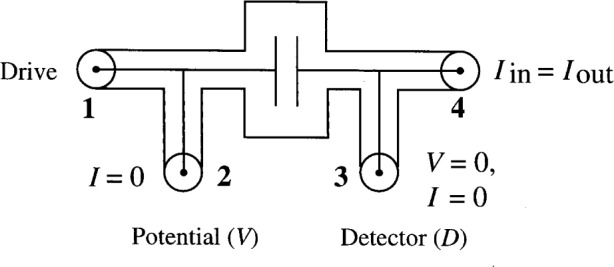
Diagram of a four-terminal-pair standard.

Four-terminal-pair measurements can be made with low uncertainty, (e.g., 0.009 μpF/pF) for a wide range of impedance values. To achieve the same results with two-terminal-pair bridges, extrapolation techniques must be used. Even then, the equivalent uncertainty can only be attained for large impedances, e.g., 10 pF to 1000 pF at 1592 Hz [1]. Four-terminal-pair measurements have the advantages of reducing the effects of parasitic impedances and diminishing the sensitivity to variations in series impedances and shunt admittances in the leads. This is especially important when measuring small impedances. Each four-terminal-pair standard has four terminal-pairs at which certain conditions must be met, as shown in Fig. 1. A terminal-pair is an associated pair of accessible terminals such as an input pair, an output pair, and the like [2]. In this case, the terminal-pair is the conductor and its shield of a coaxial connection. The four-terminal-pair standard is defined by the ratio of the open circuit voltage *V* at terminal-pair 2 to the current *I* at terminal-pair 4 (*I*_in_ = *I*_out_ at pair 4) when the current at terminal-pair 2 is zero and both the voltage and current at terminal-pair 3 are zero. This creates a well-defined standard which can be moved between different bridges and still give the same value.

The present four-terminal-pair bridge used at the National Institute of Standards and Technology (NIST) and designed by Cutkosky [3] is complex and requires a series of auxiliary balances to meet the required conditions at the four terminals of each standard. Even though combining networks are used, six auxiliary balances are needed. Furthermore, they are not totally independent; a number of iterations are required for their adjustment. The bridge described in this paper is much simpler, but still achieves the low uncertainty of the more complex four-terminal-pair bridge. What is novel about this bridge is that the auxiliary balances and the transformer that supports them are completely separate from the main bridge transformer and can easily be removed when four-terminal-pair measurements are not required.

## 2. Design

A two-terminal-pair bridge has two connections for each standard: a potential terminal and a detector terminal, whereas a four-terminal-pair bridge provides four terminal-pairs for each standard. These include a drive terminal-pair, a potential terminal-pair, an *I*_in_ = *I*_out_ terminal-pair, and a detector terminal-pair as shown in Fig. 1.

The potential terminal-pair (terminal-pair 2) supplies the potential that defines the standard and is the same as the potential terminal in a two-terminal-pair bridge. The drive terminal (terminal-pair 1) supplies current to the potential terminal-pair resulting in zero current at the potential terminal-pair. This is one of the required conditions for a four-terminal-pair measurement. No conditions are specified for the drive terminal-pair. Another condition is that the currents in the conductor and shield at the *I*_in_ = *I*_out_ terminal-pair (terminal-pair 4) are equal in magnitude and opposite in sign. The current at this terminal-pair defines the current in the standard. The detector terminal (terminal-pair 3) is the same as the detector terminal in a two-terminal-pair bridge. The current and potential at this terminal-pair must be zero, which is the last requirement for the four-terminal-pair standard.

**Fig. 2:**
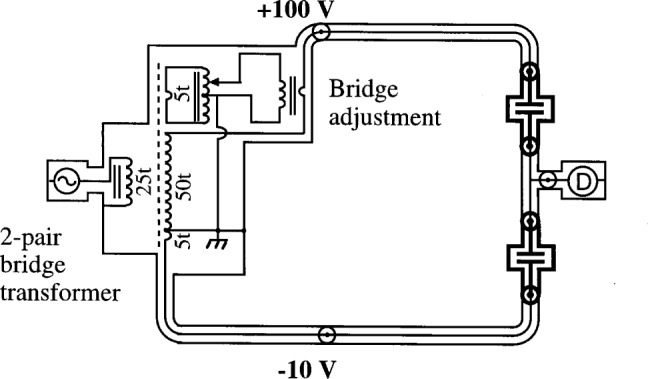
Simplified version of a 10:1 two-terminal-pair bridge. The symbol t represents the turns of the transformer. The standards are shown in bold. The coaxial chokes and the out-of-phase bridge adjustment are not shown.

**Fig. 3:**
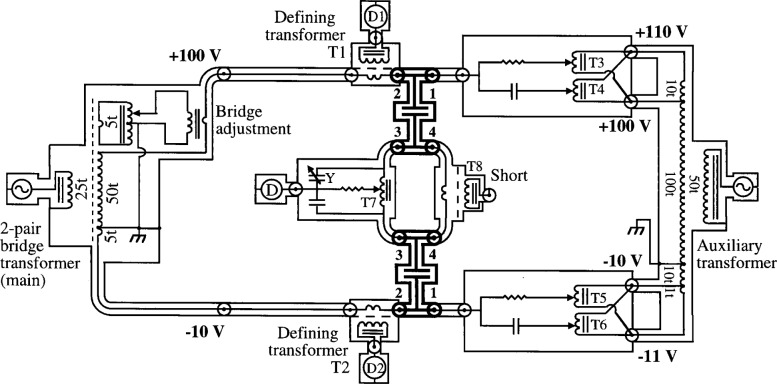
Diagram of a 10:1 ratio four-terminal-pair bridge converted from a two-terminal-pair bridge. The standards are shown in bold and the their terminals are labeled 1, 2, 3, and 4. The standards shown are capacitors, but resistors or inductors can be used as well. The symbol T refers to a transformer, D to a detector, and t represents the turns of a transformer. The coaxial chokes and the out-of-phase bridge adjustment are not shown.

The conversion to a four-terminal-pair from a two-terminal-pair bridge is achieved by providing two additional terminal-pairs (1 and 4) for each standard. The two-terminal-pair bridge is a direct reading ratio set built by Cutkosky at NIST and is similar to those described in Ref. [4]. It has half taps and tenth taps and therefore can be used as a 10:1, 2:1 or 1:1 bridge. A simplified version of a two-terminal-pair 10:1 bridge is shown in Fig. 2. The converted two-terminal-pair bridge is shown in Fig. 3. Auxiliary balances, which are described below, are used to satisfy the requirements of the four-terminal-pair definition at each terminal-pair.

Connections for the drive terminal-pairs (terminal-pair 1) are created by the addition of leads from an auxiliary transformer, which supply currents to these terminal-pairs. These leads supply current to the standard and associated cables so that there is zero current at terminal-pair 2, the potential terminal-pair. In order to provide adjustment of the currents which is done by adjusting the transformers T3, T4, T5, and T6, the voltage supply for the drive leads is at a slightly higher potential than the potential leads for the two-terminal-pair bridge. The auxiliary transformer is also built with taps so that it can be used in a 10:1, 2:1, or 1:1 ratio, sharing the same power supply as the two-terminal-pair bridge transformer (main transformer).

The condition for zero current at the potential terminal-pairs (terminal-pair 2) is met by the insertion of defining transformers T1 and T2 at these terminals. Defining transformers are used to ensure that there is zero current at the terminal-pairs and are employed with detectors (D1 and D2 in this case). A complete description of defining transformers is given in Ref. [3]. This auxiliary balance is made by adjusting T3 and T4 for a null at D1, and T5 and T6 for a null at D2, which together satisfies the condition of zero current at terminal-pair 2 of the standards. The transformers T3, T4, T5, and T6 are commercial inductive voltage dividers. The balance of D1 and D2 is very rapid since the two balances are almost independent of one another and require only one or two iterations between them. In practice, the 100 turn detector winding of each defining transformer is shorted for the main balance and a detector is connected only during the adjustment of the auxiliary balance.

The connections for terminal-pairs 4 (*I*_in_ = *I*_out_ terminals) and 3 (detector terminals) of the standards are the same as those described for Cutkosky’s four-terminal-pair bridge [3]. The 100 turn detector winding of the defining transformer T8 is kept shorted except during the adjustment of this auxiliary balance. Y and T7 are adjusted so that no change is observed at the main detector D when a voltage is injected at the 100 turn winding of T8.

## 3. Measurements

The main concern about the success of this design was whether the auxiliary balances for the drive leads would be sufficiently stable. The voltage output from a transformer will typically vary due to instabilities in the primary core, the effects of impedances in the transformer windings, and the effects of lead impedances in the cables attached to the transformer. In Cutkosky’s four-terminal-pair bridge [3], the windings that supply voltage to the auxiliary balances are wound on the same core as the windings for the main potential output; therefore variations in voltage output due to changes in its primary core and windings affect the secondary windings of both the main potential output and auxiliary potential output at the same time. In the new design, however, the auxiliary balances are supplied by a separate transformer. Hence variations in the voltage of two separate windings wound on different cores could cause instabilities in the auxiliary balances after the balance has been reached. This auxiliary balance matches the voltages from the drive lead and the potential lead at the defining transformer. If one of these voltages changes, the balance will be lost.

The possible effect of such variations on our bridge was investigated by measuring large admittances for which any voltage changes due to changes in core impedances would be magnified. The stability of the auxiliary balances was monitored over a period of time (>15 min) for a measurement of large admittances. We were able to measure admittances up to 10^−3^ S and still have no relative changes of more than 1×10^−9^ for periods longer than 15 min, which is more than adequate to obtain a bridge measurement.

The calibration of the new four-terminal-pair bridge requires the following three steps: determination of the linearity of the bridge, determination of the actual magnitude and phase angle which correspond to the changes in the real and quadrature dials of the bridge, and determination of the main transformer ratio [3]. The phase defect of the bridge should also be checked. The phase defect is the deviation from ideal in the phase of the voltage injected by the bridge adjustments. Since this four-terminal-pair bridge is made by adding only components that do not require metrological characterization to a two-terminal-pair bridge, most of these properties will be the same as those for the two-terminal-pair bridge.

### 3.1 Linearity and Magnitude

The checks on linearity and magnitude (dial settings) were done by relating the new four-terminal-pair bridge to the Cutkosky four-terminal-pair bridge of the type described in Ref. [3] These properties have already been checked for the Cutkosky bridge and found to be satisfactory. Preliminary checks on the linearity and the magnitude of the real dials were done by using the two bridges to measure the capacitance ratio of a 100 pF standard to a 1000 pF standard before and after adding 0.17 pF in parallel with the 1000 pF standard. Both bridges agreed to within the experimental relative standard uncertainty (Type A) of the measurement, which was about 3×10^−9^. The addition of 0.17 pF corresponds to a relative change of 170×10^−6^ in the bridge reading, which is a much larger deviation from nominal than any of the standards used in our present measurements. This indicates that the accuracy of the magnitude and the linearity of the new bridge are adequate for our present measurements. Since these properties depend on those of the two-terminal-pair bridge transformer which have been measured in the past and found to be satisfactory, a more rigorous check [3] of the linearity and the magnitude, is not required.

### 3.2 Transformer Ratio

The main 10:1 transformer ratio was determined using a permutation of eleven 10 pF capacitors. This method is described by Shields in Ref. [1]. For the measurement of several different standards, good agreement between the Cutkosky four-terminal-pair bridge and the new four-terminal-pair bridge was obtained. This is an additional indication that the transformer ratio is correct to within experimental uncertainty.

### 3.3 Phase Defect

The phase defect of the bridge was measured by setting up the new four-terminal-pair bridge with two resistors having known phase defect and adding capacitance in parallel with one of the resistors in order to change its phase. Since the phase dials of our two-terminal-pair bridge are limited to a range of 25 μrad, the phase changes were limited within this range. No change in the magnitude dials was observed with the phase changes, indicating no phase defect for this range.

### 3.4 Additional Tests

Another check on the new bridge was performed by comparisons against our present four-terminal-pair bridge in several different configurations for a range of impedance standards. These configurations are part of the chain of measurements in our (SI) ohm realization using the NIST calculable capacitor, and are as follows: 10 to 1 step-ups from 10 pF to 100 pF and from 100 pF to 1000 pF; 100 to 1 step-down from 100 kΩ to 1000 Ω; calibration bridge for the current transformer (100:1 resistance bridge); and calibration bridge for the main 10:1 transformer ratio. For these configurations, the new bridge agreed with Cutkosky’s four-terminal-pair bridge to within a relative difference of 2×10^−9^. These comparisons uncovered several minor problems with the Cutkosky four-terminal-pair bridge that would have otherwise gone unnoticed. These have been corrected and corresponded to relative changes of 5×10^−9^ in the measurements [5].

## 4. Conclusions

The converted four-terminal-pair bridge is much simpler to use and construct than the Cutkosky four-terminal-pair bridge [3]. It requires only three auxiliary balances and can be constructed with only additional external connections to a previously existing two-terminal-pair bridge. It has achieved a relative standard uncertainty of 1×10^−9^ for measurements over periods longer than 15 min. It was also shown to be equivalent to Cutkosky’s four-terminal-pair bridge presently used at NIST to within a relative standard uncertainty of 2×10^−9^. Two-terminal-pair standards can easily be converted to four-terminal-pair standards by the addition of two coaxial tees.

This bridge has been very useful as a check of Cutkosky’s four-terminal-pair bridge used at NIST in the (SI) ohm realization. A version of this converted bridge will also be used in quantum Hall effect ac measurements, future (SI) ohm realizations, and in the extension of the calculable capacitor to other frequencies.
